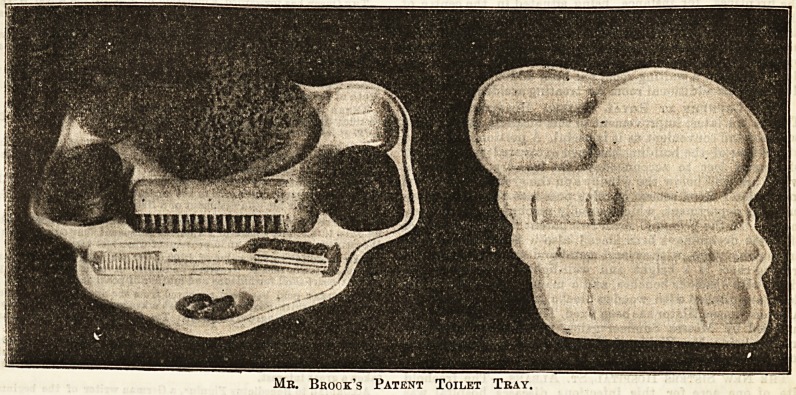# Toilet Tray

**Published:** 1893-07-08

**Authors:** 


### TOILET TRAY.

A very useful novelty has recently been brought to our notice in the shape of a small tray intended for use on the washstand, to hold all such necessaries as sponge, tooth and nail brushes, soap, &c. It is the invention of, and has been patented by Mr. Brook, of King's Langley, Herts, and has been specially designed by him to fill a want often felt in a sick-room. Nurses will appreciate the convenience of being enabled to carry to the patient's bedside all that is needed at one time in the way of such toilet accessories. Little description is needed, as our illustration, which we give by kind permission of Mr. Brook, shows clearly the uses and advantages of the trays. They may be had single or double, the prices of both being extremely moderate (about 3s.). They are manufactured in china by Messrs. Mintons, can be made to match any toilet service, and can be procured through the stores or any firm selling toilet ware. We understand they are being made in enamel and ironware as well as in china, and though the latter will undoubtedly be the most attractive in appearance, the advantages of unbreakable ware are obvious. The inventor has specially considered "the ladies" in providing a receptacle for rings, and we feel sure his tray has only to be seen to be at once taken into favour. It will be found very useful for invalids in travelling, being very compact, and thus needing little space. Quite simple in make, it is a perfectly easy matter to keep these trays clean, and Mr. Brook is to be congratulated on having produced an article which will certainly be of real service in the sick-room as well as a great convenience for ordinary use.

MR. BROOK'S PATENT TOILET TRAY.

## Figures and Tables

**Figure f1:**